# Autistic Symptoms and Social Cognition Predict Real-World Outcomes in Patients With Schizophrenia

**DOI:** 10.3389/fpsyt.2020.00524

**Published:** 2020-06-05

**Authors:** Giacomo Deste, Antonio Vita, Gabriele Nibbio, David L. Penn, Amy E. Pinkham, Philip D. Harvey

**Affiliations:** ^1^Department of Mental Health and Addiction Services, Spedali Civili Hospital, Brescia, Italy; ^2^School of Medicine, University of Brescia, Brescia, Italy; ^3^Department of Psychology, University of North Carolina, Chapel Hill, NC, United States; ^4^School of Psychology, Australian Catholic University, Melbourne, VIC, Australia; ^5^School of Behavioral and Brain Sciences, The University of Texas at Dallas, Richardson, TX, United States; ^6^Department of Psychiatry, University of Texas Southwestern Medical School, Dallas, TX, United States; ^7^Department of Psychiatry and Behavioral Sciences, University of Miami Miller School of Medicine, Miami, FL, United States; ^8^Research Service, Miami VA Healthcare System, Miami, FL, United States

**Keywords:** schizophrenia, autism spectrum disorder, social cognition, PANSS Autism Severity Score, real-world outcomes

## Abstract

**Objective:**

Real-world functioning is a complex construct influenced by different factors. The impact of social cognition and autism spectrum disorder (ASD) symptoms on different aspects of the life of people with schizophrenia has been demonstrated independently, but it is unclear how these factors are related to functioning when considered concurrently. We hypothesized that ASD symptoms could play a major role in predicting real-world functioning in schizophrenia.

**Methods:**

Existent databases from two studies (SCOPE Phase 3 and SCOPE Phase 5), in which a total of 361 patients (mean age 41.7 years; 117 females) were assessed with measures of symptom severity, neuro- and socio-cognitive abilities, functional capacity, social skills, and informant-reported real-world functioning outcomes, were analyzed.

**Results:**

Active social avoidance, social skills, ASD symptoms, and emotion processing emerged as predictors of real-world interpersonal relationships. Cognitive performance, positive symptoms, and functional capacity emerged as predictors of real-world participation in daily activities. Cognitive performance, emotion processing, positive symptoms severity, and social skills emerged as predictors of real-world work outcomes.

**Conclusion:**

Among other demographic, clinical, and functional capacity variables, increased ASD symptoms emerged as a significant predictor of poorer social relationships and may therefore represent a key factor in predicting real-world social functioning in schizophrenia.

## Introduction

### Background

The overlap between autism spectrum disorders (ASD) and schizophrenia spectrum disorders is a topic that has recently been of increasing interest and lately has been the focus of a growing body of literature, with data highlighting various similarities in pathophysiological, genetic, neuroimaging, and clinical characteristics between the two spectra (1, 2). Deficits in social interactions are considered one of the key features of ASD (3), and deficits in social cognition have been demonstrated both in patients with ASD (4, 5) and schizophrenia (6–8), with similar levels of impairment across disorders (9). Social cognition deficits have been associated also with older age, lesser education, poorer cognitive performance (10), and more severe functional impairment (11).

The presence of ASD features in patients with schizophrenia spectrum disorders has recently been investigated, leading to the development of specific instruments for the assessment of ASD symptoms in patients with schizophrenia (12). The PANSS Autism Severity Score (PAUSS) (13), a scale derived from the Positive and Negative Syndrome Scale (PANSS) (14), is one of such measures and has been shown to be able to evaluate ASD symptoms in schizophrenia patients with an accuracy comparable to that of more elaborate and time consuming tools (15). Being based on behavioral observation and symptoms severity, the PAUSS, rather than investigating “schizophrenic autism”, a construct of more experiential nature (16–18), represents a valid and practical tool for the assessment of ASD features in patients diagnosed with schizophrenia in clinical settings (15).

Recent studies show that greater severity of ASD symptoms in patients with schizophrenia spectrum disorders predicts poorer performance on different social cognitive tests, both in the emotion processing and in the mental state attribution/theory of mind domains (19). Some authors have also hypothesized that schizophrenia patients with prominent ASD symptoms may represent a subpopulation with specific clinical characteristics (12), including poorer real-world functioning and greater impairments in the ability to judge the quality of their everyday functioning (20).

Impairment in real-world settings remains one of the most problematic issues that patients with schizophrenia have to face. These deficits are likely related to difficulties in various everyday functional skills, such as initiating and maintaining social relationships, entering and maintaining paid jobs, living independently in the community, and managing self-care, health-care, and basic financial resources (21, 22). Treatments that are effective in reducing the symptoms of schizophrenia do not consistently show a parallel improvement in real-world functioning (23), and the relationship between schizophrenia symptom severity and functioning is not linear: some patients with severe symptoms may function relatively well, while others with milder symptoms may show an important functional impairment (24). Thus, in order to understand functional impairment in schizophrenia, it is likely necessary to look beyond the impact of traditional psychotic symptoms. Cognitive impairment, reduction of functional capacity, and health status, as well as other factors, appear to have a considerable impact on the everyday functioning (25, 26). In addition, recent data demonstrate that social cognition has an important influence on functional outcomes in patients with schizophrenia, and may indeed be a central factor in determining real-world outcomes (27–29), particularly when combined with consideration of both social cognitive ability and self-assessments of that ability (30). Despite the identification of these contributors, a good portion of the variance in functional outcomes remains unaccounted for (31), suggesting that the search for determinants of outcomes should continue. Given that greater ASD symptoms in schizophrenia have been independently linked to poorer social cognition and poorer functioning, it is necessary to evaluate the importance of these factors when examined concurrently with each other and with other known predictors (e.g., cognitive impairment, etc.). Doing so could yield valuable information regarding those targets that should be prioritized in treatment efforts.

### Aims of the Study

The aim of the present study was to identify the role of autism spectrum disorder symptoms, social cognitive performance, neurocognitive performance, functional capacity, and social skills in predicting real-world everyday outcomes in a sample of patients with schizophrenia spectrum disorders. Our primary hypothesis is that ASD symptoms will account for significant amounts of variance in outcomes and therefore represent an important and novel individual predictor of real-world functioning among individuals with schizophrenia. Since a possible partial overlap between negative symptoms severity and ASD features investigated by the PAUSS might be observed, active social avoidance was also included as an indirect measure of negative symptomatology, and all the analyses were performed also considering only patients with a low level of active social avoidance (PANSS G16 ≤ 3).

## Methods

### Participants

Data analyzed in the present study were obtained by merging the datasets originally elaborated for two previously published studies (32, 33). In both of these studies, patients diagnosed with schizophrenia or schizoaffective disorders were assessed with clinical, psychosocial, neuro-cognitive and social-cognitive measures. These studies are part of a larger research project, the Social Cognition Psychometric Evaluation (SCOPE). Data included from the first dataset were gathered from the third phase of the whole research project, SCOPE Phase 3 (32), while data included from the second dataset were gathered for the fifth and final part of the project, SCOPE Phase 5 (33). For the SCOPE 3 study a total of 179 patients were recruited at two sites, the Southern Methodist University and the Miami Miller School of Medicine, while the SCOPE 5 study included a total of 218 patients recruited at three sites, the University of Texas at Dallas, the University of Miami Miller School of Medicine, and the University of North Carolina at Chapel Hill.

Both studies used identical inclusion and exclusion criteria. To be included in the studies, patients had to satisfy the following criteria: (I) diagnosis of Schizophrenia or Schizoaffective Disorder, according to the Diagnostic and Statistical Manual of Mental Disorders, Fourth Edition (DSM-IV) (34), confirmed with the Mini International Neuropsychiatric Interview (35) and the Structured Clinical Interview for DSM Disorders Psychosis Module (36), (II) patients had to be clinically stable, without any hospitalization occurring in the previous two months, without any change in the medication regimen for a minimum of 6 weeks, and without any medication dosage change for a minimum of 2 weeks.

Patients were excluded from the studies if they presented one of the following: (I) presence or history of pervasive developmental disorder, including ASD, or of mental retardation with an IQ < 70, as defined by the diagnostic criteria reported in the DSM-IV, (II) presence or history of any medical or neurological illness that could have a negative impact on the functioning of the central nervous system, including epilepsy and seizures, neoplasms of the central nervous system structures, inflammatory or autoimmune disorders affecting the central nervous system, (III) presence of visual or hearing impairment severe enough to limit the participation of the patients in the assessment, (IV) no or very limited proficiency with English language, (V) presence of substance abuse in the past month, (VI) presence of active substance dependence in past six months.

A number of different tests were administered to asses social cognition in the SCOPE 3 and the SCOPE 5 studies. Only the measures of social cognition included in both studies were utilized in the current analyses. Additionally, a small number of patients participated in both SCOPE 3 and SCOPE 5. To maintain the independence of the data, any SCOPE 5 data from repeat participants were removed from the final sample. The resulting sample size is therefore smaller than the sum of the number of patients recruited in the two SCOPE studies.

### Measures

#### Social Cognition

For the assessment of social cognition, the following tests, used in both SCOPE 3 and SCOPE 5, were used for this study and included in the analyses. The *Bell Lysaker Emotion Recognition Task* (BLERT) (37) is a test measuring the ability to recognize seven basic emotional states: happiness, sadness, fear, disgust, surprise, anger, or no emotion. A series of 21 short video clips, presenting a male actor providing dynamic facial, vocal-tonal, and upper-body movement cues, is presented to the participant, who has to identify the emotion represented by the actor after each videoclip. The total number of correctly identified emotions represents the final score, ranging from 0 to 21.

The *Penn Emotion Recognition Text* (ER-40) (38) is a test composed of 40 color photographs of static faces. Each picture expresses one of four basic emotions (happiness, sadness, anger, or fear) or a neutral expression, and are balanced for model's sex, age, and ethnicity. For each emotion, four high-intensity and four low-intensity expressions are provided. The total number of correctly identified emotions represents the final score, ranging from 0 to 40.

The *Reading the Mind in the Eyes Test* (Eyes) (39) is a test designed to assess the ability to understand the mental state of other people form the expression of the eye region of the face. A set of 36 pictures of the eye region of different faces is presented to the participants, who have to identify the mental state presented among four different choices. The number of correct answers represents the final score, ranging from 0 to 36.

The *Awareness of Social Inferences Test, Part 3* (TASIT) (40) is a test assessing the capacity to detect and identify social exchanges, such as lies and sarcasm. Participants are shown a series of videos representing everyday social interactions and have to answer four standardized question concerning the intentions, beliefs, and meaning of the speakers and their interactions. The number of correct answers represents the final score, ranging from 0 to 64.

The *Hinting Task* (Hinting) (41) is a test assessing the ability to infer the true intent of indirect speech. Ten short passages, depicting an interaction between two fictional characters, are read by the experimenter, and each passage ends with one of the two characters dropping a hint to the other one. The participant is asked to explain what the character dropping the hint truly meant. If an incorrect answer is provided by the participant, a second hint is presented, allowing the possibility to earn partial credit. The total score ranges from 0 to 20.

#### Neurocognition

The neurocognitive assessment was composed of a subset of the tests from the MATRICS Consensus Cognitive Battery (MCCB) (42): Trail Making Test—Part A (TMT-A); BACS Symbol Coding; BACS Category Fluency (Animal Naming); Letter–Number Span; and Hopkins Verbal Learning Test—Revised (HVLT-R). These test are designed to evaluate different cognitive domains that appear to be impaired in patients with schizophrenia (43) such as processing speed, working memory, and verbal memory (44–46). Following the recommendation of the developers of the battery, a global composite score was calculated by averaging the t-scores of all the tests (47). Patients' premorbid IQ was also taken into account and was assessed with the Wide Range Achievement Test-3 Reading subscale (WRAT-3) (48). The WRAT-3 Standard Score was included in the analyses.

#### Clinical Symptoms

All patients were evaluated with the Positive And Negative Syndrome Scale (PANSS) (14). The PANSS Autism Severity Score (PAUSS), a scale derived from the PANSS for the measure of autistic symptoms in patients with schizophrenia (13), was used and included in the analyses. The PAUSS is calculated by summing eight different PANSS items: N1 (“blunted affect”), N3 (“poor rapport”), N4 (“social withdrawal”), N5 (“difficulties in abstract thinking”), N6 (“lack of spontaneity and flow of conversation”), N7 (“stereotyped thinking”), G5 (“mannerism”), and G15 (“preoccupation”).

PANSS item G16, “active social avoidance”, was also used and included in the analyses as a measure of potential social deficits. This PANSS item does not contribute to the PAUSS total score.

The PANSS positive subscale was also used and included in the analyses, based on the previous results of analyses of the PANSS (49). The PANSS positive subscale is composed of the sum of the following items: P1 (“delusions”), P2 (“conceptual disorganization”), P3 (“hallucinations”), P4 (“excitement”), P5 (“grandiosity”), P6 (“suspiciousness/persecution”) and P7 (“hostility”).

#### Functional Skills

Functional capacity was assessed with the UCSD Performance-Based Skills Assessment, Brief (UPSA-B) (50). The UPSA-B is a brief and widely used scale that assesses financial and communication skills involved in community tasks. The total score ranges from 0 to 100.

Social competence was assessed with the Social Skills Performance Assessment (SSPA) (51). The SSPA is a role-play measure in which participants have to start and maintain a conversation in two different social situations: meeting a new neighbor and negotiating with a landlord to fix a leak. Roleplay sessions are recorded and coded by an expert rater blind to participant diagnosis. The following variables are rated during the evaluation: interest, fluency, clarity, focus, overall abilities, and social appropriateness for both sessions, and negotiation ability and persistence are also rated for the landlord session only. The final total score is the mean score across both role-plays, ranging from 1 to 5.

#### Real-World Outcomes

Finally, real-world functional outcomes were assessed with the Specific Level of Functioning Scale (SLOF) (52). The SLOF is an informant-rated measure and, in its 24-item form, is composed of three subscales, one for social functioning (interpersonal relationships) and two for community-living skills (participation in activities and work skills). Informants were identified by the participants and were either high-contact clinicians, close friends, or family members. Each item is rated from 1 to 5, with higher scores indicating better functioning, and the final score of every subscale is the mean of the scores of every item composing the scale. The SLOF has been found to be a reliable and valid instrument to assess real-world functioning in patients with schizophrenia, with good construct validity and internal consistency (22, 53).

### Statistical Analysis

To identify predictors of real-world functional outcome, multivariate linear regression analyses were performed, including the Interpersonal Relationships, Activities, and Work subscales of the SLOF as dependent variables, and demographic variables, PANSS positive symptoms score, PAUSS total score, PANSS item G16 (“Active Social Avoidance”), social cognitive performance, global cognitive composite score, UPSA-B score, and SSPA score as potential predictors. Potential predictors were included in the regression if they were found to be significant in univariate exploratory analyses, performed by correlating continuous variables with the dependent variables, and by using t-test for dichotomous potential predicting variables (*i.e.*, sex). Parametric statistics were adopted regardless of the distribution of the data due to the large size of the investigated sample. This conservative approach was used in order to avoid false negatives (or type II errors) (54, 55). Multiple linear regressions were conducted using a stepwise procedure. As the number of potential predictors in each model was lower than one for every twenty observed subjects, the number of the included predictors was considered appropriate (56, 57).

As active social avoidance has been shown to be a strong predictor of social competence and social functioning in patients with schizophrenia (28, 58), high levels of active social avoidance have been considered a possible confounder that could override the influence of other variables in the analyses. Therefore, all the analyses were also performed on a subgroup of the whole sample composed of patients with a score on PANSS G16 (“active social avoidance”) ≤3, numbering a total of 296 patients and representing 81% of the total sample.

Statistical analyses were performed using SPSS 15.0 software. P-values < 0.05 (2-tailed) were considered significant.

## Results

### Full Sample

#### Correlational Analyses

The characteristics of the sample are presented in **Table 1**. The univariate correlations are shown in **Table 2**. Potential predictors of better real-world interpersonal relationships outcomes (as measured by SLOF: Interpersonal relationships subscale) that emerged at the univariate analyses were: more education years, higher premorbid IQ (WRAT-3 standard score), better neurocognitive performance (Neurocog), better functional capacity (UPSA-B), better social skills (SSPA), less severe ASD features (PAUSS), less severe positive symptoms (PANSSpos), less severe active social avoidance (PANSS-G16), better emotion processing performance (as measured by both BLERT and ER-40), and better theory of mind (TASIT).

**Table 1:** Characteristics of the Sample.

Variable	N, Mean ± SD
N	361
M:F	244:117
Age (Years)	41.7 ± 12.0
Education (Years of Education)	12.9 ± 2.3
WRAT-3 Standard Score (Premorbid IQ)	94.4 ± 15.2
PAUSS total score (Autism)	15.3 ± 5.4
PANSS Positive Score (Positive Symptoms Severity)	16.14 ± 5.4
Active Social Avoidance (PANSS-G16)	2.2 ± 1.4
Global Cognitive Composite Score (t-score)	38.2 ± 7.1
UPSA-B (Functional Capacity)	69.7 ± 14.1
SSPA (Social Skills)	4.1 ± 0.5
BLERT (Social Cognition–Emotion Processing)	13.5 ± 4.0
ER-40 (Social Cognition–Emotion Processing)	30.2 ± 5.1
EYES (Social Cognition–Mental State Attribution)	20.6 ± 5.4
HINTING (Social Cognition–Mental State Attribution)	13.3 ± 3.8
TASIT (Social Cognition–Mental State Attribution)	44.4 ± 7.5

**Table 2:** Correlations between SLOF scales and demographic, cognitive, clinical, and social cognitive variables.

Variables	SLOF Inf: Interpersonal Relationships	SLOF Inf: Activities	SLOF Inf: Work
Age	−0.053 (p = 0.021)	0.023 (p = 0.666)	−0.142** (p =0.007)
Education Years	0.143** (p = 0.007)	0.160** (p = 0.002)	0.225** (p = < 0.001)
Premorbid IQ (WRAT-3 Standard Score)	0.121* (p = 0.022)	0.120* (p = 0.023)	0.236** (p = < 0.001)
Neurocog	0.191** (p = < 0.001)	0.248** (p = < 0.001)	0.344** (p = < 0.001)
UPSA-B	0.155** (p = 0.005)	0.219** (p = < 0.001)	0.293** (p = < 0.001)
SSPA	0.286** (p = < 0.001)	0.176** (p = 0.001)	0.287** (p = < 0.001)
PAUSS	−0.387** (p = < 0.001)	−0.114* (p = 0.031)	−0.184** (p = < 0.001)
PANSSpos	−0.204** (p = < 0.001)	−0.149** (p = 0.005)	−0.191** (p = < 0.001)
PANSS-G16	−0.414** (p = < 0.001)	−0.104* (p = < 0.049)	−0.095 (p = < 0.071)
BLERT	0.221** (p = < 0.001)	0.140** (p = 0.008)	0.303** (p = < 0.001)
ER40	0.122* (p = 0.021)	0.119* (p = 0.024)	0.181** (p = 0.001)
EYES	0.103 (p = 0.052)	0.076 (p = 0.149)	0.218** (p = < 0.001)
HINTING	0.095 (p = 0.073)	0.122* (p = 0.021)	0.203** (p = < 0.001)
TASIT	0.145** (p = 0.006)	0.159** (p = 0.003)	0.327** (p = < 0.001)

Pearson's r (p values).

*p < 0.05.

**p < 0.01.

Potential predictors of better real-world participation in daily activities outcomes (as measured by SLOF: Activities subscale) that emerged at the univariate analyses were: more education years, higher premorbid IQ (WRAT-3 standard score), better neurocognitive performance (Neurocog), better functional capacity (UPSA-B), better social skills (SSPA), less severe ASD features (PAUSS), less severe positive symptoms (PANSSpos), less severe active social avoidance (PANSS-G16), better emotion processing performance (as measured by both BLERT and ER-40), and better theory of mind (as measured by both HINTING and TASIT).

Potential predictors of better real-world work outcomes (as measured by SLOF: Work subscale) that emerged at the univariate analyses were: younger age, more education years, higher premorbid IQ (WRAT-3 standard score), better neurocognitive performance (Neurocog), better functional capacity (UPSA-B), better social skills (SSPA), less severe ASD features (PAUSS), less severe positive symptoms (PANSSpos), better emotion recognition skills (as measured by both BLERT and ER-40) and better theory of mind (as measured by EYES, HINTING and TASIT). All statistically significant relationships across the three outcomes represented small to medium effect sizes.

No differences between males and females emerged in any of the SLOF subscales analyzed at the univariate t-tests (**Table 3**).

**Table 3:** Functional outcome differences between male and female patients (t-test).

SLOF scale	Males (mean ± SD)	Females (mean ± SD)	t-test p	Cohen's d
**SLOF: Interpersonal Relationships**	3.32 ± 0.84	3.33 ± 0.91	0.891	0.011
**SLOF: Activities**	4.52 ± 0.82	4.49 ± 0.79	0.758	0.037
**SLOF: Work**	3.72 ± 0.82	3.75 ± 0.83	0.738	0.036

#### Regression Analyses

**Table 4** shows the individual predictors of real-world functional outcomes. Individual predictors of real-world social functioning, as measured by the SLOF Interpersonal Relationships subscale, were less severe Active Social Avoidance (PANSS-G16) (p < 0.001), better social skills (SSPA) (p = 0.011), less severe ASD symptoms (PAUSS) (p < 0.001), and better emotion processing performance (BLERT) (p = 0.001) (Model: F = 33.755, R*^2^* = 0.290, p < 0.001).

**Table 4:** Predictors of functional outcome (Stepwise linear multivariate regressions).

Dependent variables	Individual predictors	Standardized Beta	T	P	Adj.R^2^	Adj. R^2^ Change
**SLOF:** **Interpersonal Relationships**	PANSS-G16	−0.354	−7.276	<0.001	0.164	
	SSPA	0.138	2.552	0.011	0.236	0.072
	PAUSS	−0.189	−3.526	<0.001	0.261	0.025
	BLERT	0.158	3.210	0.001	0.281	0.020
	*Model F = 33.755, R^2^ = 0.290, Adj. R^2^ = 0.281*			<0.001		
**SLOF:** **Activities**	Neurocog	0.183	3.005	0.003	0.061	
	PANSSpos	−0.116	−2.195	0.029	0.075	0.014
	UPSA-B	0.127	2.071	0.039	0.084	0.009
	*Model F = 11.267, R^2^ = 0.093, Adj. R^2^ = 0.084*			<0.001		
**SLOF:** **Work**	Neurocog	0.221	3.941	<0.001	0.119	
	BLERT	0.192	3.464	0.001	0.158	0.039
	PANSSpos	−0.166	−3.343	0.001	0.187	0.029
	SSPA	0.111	2.023	0.044	0.195	0.008
	*Model F = 21.224, R^2^ = 0.205, Adj. R^2^ = 0.195*			<0.001		

Real-world community living skills, as measured by the SLOF Activities subscale, were predicted by better global cognitive performance (Neurocog) (p = 0.003), less severe positive symptoms severity (PANSSpos) (p = 0.029), and better functional capacity (UPSA-B) (p= 0.039) (Model: F = 11.267, R*^2^* = 0.093, p < 0.001).

Finally, real-world work outcomes, as measured by the SLOF Work subscale, were predicted by better global cognitive performance (Neurocog) (p < 0.001), better emotion processing performance (BLERT) (p = 0.001), less severe positive symptoms (PANSSpos) (p = 0.001), and better social skills (SSPA) (p = 0.044) (Model: F = 21.224, R*^2^* = 0.205, p < 0.001).

### Low Active Social Avoidance Subsample

#### Correlational Analyses

Univariate correlations for patients with low active social avoidance, as identified by PANSS item G16 (PANSS-G16 ≤ 3; n = 296), are shown in **Table 5**. In this subgroup of patients, potential predictors of better real-world interpersonal relationships outcomes (as measured by SLOF: Interpersonal relationships subscale) that emerged at the univariate analyses were: more education years, better neurocognitive performance (Neurocog), better functional capacity (UPSA-B), better social skills (SSPA), less severe ASD features (PAUSS), less severe positive symptoms (PANSSpos), less severe active social avoidance (PANSS-G16), better emotion processing performance (as measured by both BLERT and ER-40), and better theory of mind (as measured by EYES, HINTING and TASIT).

**Table 5:** Correlations between SLOF scales and demographic, cognitive, clinical, and social cognitive variables in patients with Low PANSS-G16 (3 or lower).

Variables	SLOF Inf: Interpersonal Relationships	SLOF Inf: Activities	SLOF Inf: Work
Age	−0.025 (p = 0.671)	0.068 (p = 0.245)	−0.109 (p =0.062)
Education Years	0.133* (p = 0.023)	0.119* (p = 0.042)	0.225** (p = < 0.001)
Premorbid IQ (WRAT-3 Standard Score)	0.083 (p = 0.157)	0.061 (p = 0.301)	0.223** (p = < 0.001)
Neurocog	0.231** (p = < 0.001)	0.232** (p = < 0.001)	0.345** (p = < 0.001)
UPSA-B	0.164** (p = 0.006)	0.202** (p = 0.001)	0.279** (p = < 0.001)
SSPA	0.323** (p = < 0.001)	0.201** (p = 0.001)	0.291** (p = < 0.001)
PAUSS	−0.419** (p = < 0.001)	−0.142* (p = 0.015)	−0.164** (p = 0.005)
PANSSpos	−0.154** (p = 0.008)	−0.144* (p= 0.014)	-0.144* (p= 0.014)
PANSS-G16	−0.292** (p = < 0.001)	−0.047 (p = 0.418)	0.056 (p = 0.339)
BLERT	0.255** (p = < 0.001)	0.124* (p = 0.034)	0.304** (p = < 0.001)
ER40	0.171* (p = 0.003)	0.125* (p = 0.033)	0.189** (p =0.001)
EYES	0.124* (p = 0.033)	0.081 (p = 0.166)	0.222** (p = < 0.001)
HINTING	0.151* (p = 0.010)	0.104 (p = 0.076)	0.190** (p = < 0.001)
TASIT	0.172** (p = 0.003)	0.146* (p = 0.012)	0.313** (p = < 0.001)

Pearson's r (p values). *p < 0.05.

**p < 0.01.

In patients with low active social avoidance, potential predictors of better real-world participation in daily activities outcomes (as measured by SLOF: Activities subscale) that emerged at the univariate analyses were: more education years, better neurocognitive performance (Neurocog), better functional capacity (UPSA-B), better social skills (SSPA), less severe ASD features (PAUSS), less severe positive symptoms (PANSSpos), better emotion processing performance (as measured by both BLERT and ER-40), and better theory of mind (TASIT).

In patients with low active social avoidance, potential predictors of better real-world work outcomes (as measured by SLOF: Work subscale) that emerged at the univariate analyses were: more education years, higher premorbid IQ (WRAT-3 standard score), better neurocognitive performance (Neurocog), better functional capacity (UPSA-B), better social skills (SSPA), less severe ASD features (PAUSS), less severe positive symptoms (PANSSpos), better emotion processing performance (as measured by both BLERT and ER-40), and better theory of mind (as measured by EYES, HINTING and TASIT). As in the full sample, all statistically significant relationships across the three outcomes represented small to medium effect sizes.

No differences between males and females emerged in any of the SLOF subscales analyzed at the univariate t-tests in patients with low active social avoidance (**Table 6**).

**Table 6:** Functional outcome differences between male and female patients (t-test) in patients with Low PANSS-G16 (3 or lower).

SLOF scale	Males (mean ± SD)	Females (mean ± SD)	t-test p	Cohen's d
**SLOF: Interpersonal Relationships**	3.42 ± 0.83	3.55 ± 0.89	0.236	0.151
**SLOF: Activities**	4.56 ± 0.81	4.51 ± 0.80	0.576	0.062
**SLOF: Work**	3.78 ± 0.83	3.79 ± 0.86	0.984	0.011

#### Regression Analyses

**Table 7** shows the individual predictors of real-world functional outcome in this subgroup of patients. In patients with low active social avoidance, individual predictors of real-world social functioning, as measured by the SLOF Interpersonal Relationships subscale, were less severe ASD symptoms (PAUSS) (p < 0.001), better emotion processing performance (BLERT) (p < 0.001), less severe Active Social Avoidance (PANSS-G16) (p < 0.001), better social skills (SSPA) (p = 0.037) (Model: F = 22.473, R*^2^* = 0.251, p < 0.001).

**Table 7:** Predictors of functional outcome in patients with Low PANSS-G16 (3 or lower) (Stepwise linear multivariate regressions).

Dependent variables	Individual predictors	Standardized Beta	T	P	Adj. R^2^	Adj. R^2^ Change
**SLOF:** **Interpersonal Relationships**	PAUSS	−0.249	−3.890	<0.001	0.165	
	BLERT	0.201	3.576	<0.001	0.206	0.041
	PANSS-G16	−0.191	−3.304	0.001	0.230	0.024
	SSPA	0.129	2.094	0.037	0.240	0.010
	*Model F = 22.473, R^2^ = 0.251, Adj. R^2^ = 0.240*			<0.001		
**SLOF:** **Activities**	Neurocog	0.233	3.970	<0.001	0.052	
	PANSSpos	−0.133	−2.267	0.024	0.066	0.012
	*Model F = 10.579, R^2^ = 0.073, Adj. R^2^ = 0.066*			<0.001		
**SLOF:** **Work**	Neurocog	0.257	4.285	<0.001	0.120	
	BLERT	0.230	3.828	<0.001	0.162	0.042
	PANSSpos	−0.141	−2.558	0.011	0.179	0.017
	*Model F = 20.702, R^2^ = 0.188, Adj. R^2^ = 0.179*			<0.001		

In patients with low active social avoidance, real-world community living skills, as measured by the SLOF Activities subscale, were predicted by better global cognitive performance (Neurocog) (p < 0.001), and less severe positive symptoms severity (PANSSpos) (p = 0.024) (Model: F = 10.579, R*^2^* = 0.073, p < 0.001).

Finally, in this subgroup of patients, real-world work outcomes, as measured by the SLOF Work subscale, were predicted by better global cognitive performance (Neurocog) (p < 0.001), better emotion processing performance (BLERT) (p < 0.001) and less severe positive symptoms (PANSSpos) (p = 0.011) (Model: F = 20.720, R*^2^* = 0.188, p < 0.001).

## Discussion

The current study examined multiple potential predictors of real-world functioning in schizophrenia spectrum disorders and specifically sought to determine whether increased ASD symptoms would emerge as an independent and important contributor to functioning even when considering the influence of well-established predictors such as cognition and social cognition. Different clinical, neurocognitive, and social-cognitive variables emerged as individual predictors of real-world functioning, with specific factors predicting different functioning outcomes.

In particular, ASD symptoms emerged as predictors of real-world social functioning. This finding confirms the hypothesis that increased ASD symptoms could play an important role in the poorer social relationships experienced by patients with schizophrenia spectrum disorders, not only for their influence on social cognitive abilities, as previously demonstrated (19), but also as an independent individual predictor. In contrast, ASD symptoms did not emerge as individual predictors of real-life community living skills or working skills in the regression analyses despite emerging as potential predictors in the univariate analyses. This suggests a stronger impact of ASD symptoms on interpersonal relationships and social outcomes, than on other real-world functioning areas that may be less socially relevant.

Lower active social avoidance, as measured by the PANSS item G-16, emerged as another individual predictor of real-world social functioning, but not of real-life community living skills or working skills. The decision to use this PANSS item, was due to the fact that it's included in the PANSS negative subscale derived from PANSS factor analysis (49), but not in the PAUSS (13), so it was used as a measure of negative symptomatology, outside ASD symptoms. This helped to further corroborate the notion of a specific predicting role of ASD symptoms on interpersonal relationship skills.

Emotion processing, a domain of social cognition, as measured by the BLERT, emerged as a predictor of both social functioning and working skills. This finding confirms the hypothesis regarding the role of social cognition in influencing real-world functioning in patients with schizophrenia (27). In particular, deficits in social cognition appear to impact not only social functioning, but also working performance, a fact already demonstrated (29).

Moreover, among the different social cognitive measures employed, assessing the domains of both emotion processing and mental state attribution, only the BLERT task (emotion processing) emerged as a predictor of functional outcomes. This also confirms the utility of the BLERT among the different tests available for the assessment of social cognitive abilities in patients with schizophrenia: indeed, the SCOPE studies already identified the BLERT as one of the key social cognitive tasks available and recommended its use in the context of clinical research, as it also shows good test–retest reliability, limited potential for floor and ceiling effects, high practicality, and good tolerability for the patient (32, 33).

Global cognitive performance was linked to community-living outcomes, both in the participation of activities and in working skills. This finding is in line with those already reported in literature (25). However, global neurocognitive performance did not predict real-world social functioning, further underlying the notion that specific functioning domains are differentially predicted by neuro- and social-cognitive abilities. In a similar way, positive symptom severity predicted both work abilities and participation in daily activities, but not social outcomes, highlighting a more specific impact of positive symptom severity on less social real-world functioning outcomes.

Social skills, as evaluated by the roleplay in the SSPA, predicted both real-world social and working outcomes, whereas functional capacity (UPSA-B) predicted real-world participation in activities only. The role of social skills in determining real-world outcomes has been already demonstrated (28) as has the link between functional capacity and real-world community-living skills (21). The fact that both social skills and social cognition emerged as predictors not only of social, but also of working outcomes underlines the impact of these abilities on different aspects of functioning and may thus represent a key factor determining global outcomes in patients with schizophrenia.

When comparing the different linear regression models, more variance was explained for interpersonal relationships (Adjusted R^2^ = 0.281) than participation in daily activities (Adjusted R^2^ = 0.084) and working skills (Adjusted R^2^ = 0.195). This could be due to a more direct relationship between social functioning and various explored potential predictors, in particular ASD symptoms and social cognitive performance. A considerable portion of variance remained to be explained, suggesting that other factors besides those investigated in this study could contribute to real-world outcomes. However, this was an expected observation, as real-world functioning is a complex construct to which many different elements may contribute (25).

Considering patients with low levels of active social avoidance, predictors of real-world social functioning did not differ in general from those found in the analysis performed on the whole sample. However, in these patients, representing a large proportion of the sample (81%), ASD features and social cognitive performance showed an even more prominent role as predictors, underlining their important function as determinants of real-world social functioning, when taking into account negative symptomatology.

This study has limitations. The social cognition measures available measured only two of the four recognized domains of social cognition, with no tasks assessing attributional style/bias and social perception. The psychometric proprieties of available measures for these two social cognitive domains are not completely satisfactory, and no test is currently recommended for use in clinical research settings (33). Moreover, attributional style also emerged as separate from other socio-cognitive skills in subjects diagnosed with schizophrenia in a recent factor analysis (59).

The SCOPE studies, from which the database for this study was derived, were not designed and conducted with the specific objective of assessing the role of potential predictors in determining real-world outcomes, or to specifically investigate ASD features in patients with schizophrenia spectrum disorders. Therefore, some aspects of global real-world functioning, included in the SLOF, such as physical functioning or self-care skills, were not assessed.

Finally, as the PAUSS scale includes various items from the PANSS negative subscale, only indirect measures of negative symptomatology, such as active social avoidance, could be included in the analyses. Further differentiating the role of negative symptoms severity and ASD features in influencing cognitive and functional outcomes of schizophrenia patients represents an important issue that should be addressed in future studies.

Nonetheless, the findings of the present study support the role of ASD features in influencing real-world social outcomes in patients with schizophrenia spectrum disorders, demonstrating that increased ASD symptoms are related to poorer social outcomes. The current findings also confirm the central role of social cognition in determining different aspects of global functioning. Future research should continue to explore the impact of ASD symptoms in patients with schizophrenia spectrum disorder, considering the hypothesis that patients with prominent ASD features could represent a particular subpopulation, with specific clinical, neuro- and social cognitive and functioning characteristics, with possible specific illness trajectories.

## Data Availability Statement

The datasets used in this study are available from Dr. Philip Harvey at the following e-mail address: pharvey@miami.edu.

## Ethics Statement

The studies involving human participants were reviewed and approved by the ethics Committees at the University of Miami, University of Texas at Dallas, and the University of North Carolina at Chapel Hill. The University of Miami IRB has agreed the current analyses are exempt from review as human subjects because of the de-identified nature of the data. The patients/participants provided their written informed consent to participate in this study.

## Author Contributions

PH designed the project, reviewed and discussed the data and statistical analyses and the final version of the paper. AP and DP participated in the design of the study. GN prepared the database and participated in the analyses. GD participated in the analyses and wrote the paper. AV participated in the design of the project and discussion of the data and manuscript. All authors contributed to and approved the final manuscript.

## Funding

This research was funded by Grant number RO1MH093432 to PH, AP, and DP from the US National Institute of Mental Health.

## Conflict of Interest

In the last year PH has received consulting fees or travel reimbursements from: Alkermes, Boehringer Ingelheim, Intra-Cellular Therapies, Jazz Pharma, Lundbeck Pharma, Minerva Pharma, Otsuka America (Otsuka Digital Health), Sanofi Pharma, Sunovion Pharma, and Teva. He receives royalties from the Brief Assessment of Cognition in Schizophrenia and the MATRICS Consensus Battery. He has a research grant from Takeda and from the Stanley Medical Research Foundation. In the last two years AV has received directly or indirectly support for clinical studies or trials, conferences, consultancies, congress presentations, advisory boards from: Angelini, Boheringer Ingelheim, Eli Lilly, Fidia, Forum Pharmaceutical, Innovapharma, Janssen-Cilag, Lundbeck, Otsuka, Recordati, and Takeda. In the last year, AP has received consulting fees and travel reimbursements from Roche.

The remaining authors declare that the research was conducted in the absence of any commercial or financial relationships that could be construed as a potential conflict of interest.
